# Trends in summer bottom-water temperatures on the northern Gulf of Mexico continental shelf from 1985 to 2015

**DOI:** 10.1371/journal.pone.0184350

**Published:** 2017-09-07

**Authors:** R. Eugene Turner, Nancy N. Rabalais, Dubravko Justić

**Affiliations:** 1 Department of Oceanography and Coastal Sciences, Louisiana State University, Baton Rouge, Louisiana, United States of America; 2 Louisiana Universities Marine Consortium, Chauvin, Louisiana, United States of America; Universidade de Aveiro, PORTUGAL

## Abstract

We quantified trends in the 1985 to 2015 summer bottom-water temperature on the northern Gulf of Mexico (nGOM) continental shelf for data collected at 88 stations with depths ranging from 3 to 63 m. The analysis was supplemented with monthly data collected from 1963 to 1965 in the same area. The seasonal summer peak in average bottom-water temperature varied concurrently with air temperature, but with a 2- to 5-month lag. The summer bottom-water temperature declined gradually with depth from 30 ^o^C at stations closest to the shore, to 20 ^o^C at the offshore edge of the study area, and increased an average 0.051 ^o^C y^-1^ between1963 and 2015. The bottom-water warming in summer for all stations was 1.9 times faster compared to the rise in local summer air temperatures, and 6.4 times faster than the concurrent increase in annual global ocean sea surface temperatures. The annual rise in average summer bottom-water temperatures on the subtropical nGOM continental shelf is comparable to the few published temperature trend estimates from colder environments. These recent changes in the heat storage on the nGOM continental shelf will affect oxygen and carbon cycling, spatial distribution of fish and shrimp, and overall species diversity.

## Introduction

The earth’s climate changed in various ways, amounts and intervals through geologic time, and recently from the added influence of changes in carbon storage on a planetary scale [1,2,3,4]. The excess in global heat content is presently being stored mostly in the upper ocean (300 to 1000 m) [5,6]. Its distribution in the open ocean varies horizontally and vertically in response to currents, and to fluctuations in solar insolation, clouds, and various atmospheric factors, among others, that are regionally diverse [1]. However, the direct and long-term quantification of global temperature changes on continental shelves is not well-represented in the scientific literature because of, for example, their small area relative to the open ocean, insufficient monitoring efforts, and/or funding limitations. There are longer-term analyses from satellites whose sensors are restricted to the surface layer so that systematic measurements of subsurface temperature values have only been carried out for the last few decades [7,8,9]. Direct and deeper temperature observations are important data acquisitions necessary to improve proxy measurements and for accurate assessments using models.

The US continental shelves are areas of intense resource exploitation and home to 39% of the US population [10]. The known effects of temperature rise on continental shelves includes influencing the timing of the spring phytoplankton blooms [11], fish community re-distributions [12,13], organism size [14], and marine biodiversity [15]. Improving our information on the continental shelf temperature trends into the assessment of the Earth’s changing heat budget, therefore, has important conservation and management implications.

Recent changes in the bottom-water temperature have been documented in the Bornholm Basin, Gotland Basin, and Baltic Sea [16] and the North Sea [8]. We know of no published analyses of decades-long temperature records for the continental shelf of the northern Gulf of Mexico (nGOM). About a third of the GOM shelf (38%) is <20 m deep and consists of shallow and intertidal area, 22% is an intermediate region from 20 to 180 m deep, 22% is the slope region from 180 to 3,000 m, and the remainder is the abyssal plain [17]. Our main objective here is to develop a piece of this missing information by analyzing a long-term data record for the northern GOM continental shelf that was conducted for a hypoxia (low-oxygen) research program starting in 1985 [18]. The sampling occurred mostly in the second half of July beginning in 1985, and included the area from the Mississippi River to the Texas-Louisiana border and occasionally further southward along the Texas coast. The data were examined to determine possible trends in the summer bottom-water temperature from 1985 to 2015 and compared to published data collected in 1963 to 1965, and also to determine trends in five different depth zones. The influence of local air temperature on the seasonal and annual variations in the bottom-water temperature were also assessed.

## Materials and methods

### Study area

**Fig 1:**
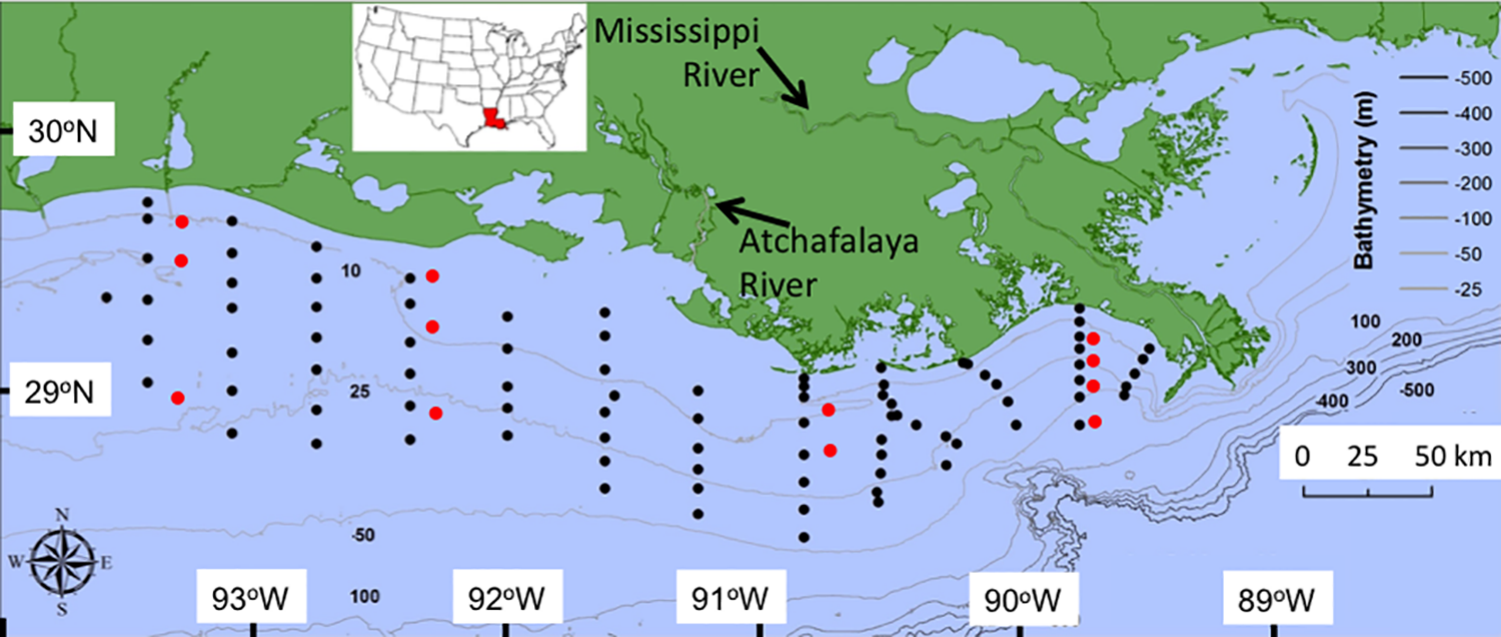
Station locations. The black dots are stations from 1985 to 2015, and the red dots are from 1963–64.

The microtidal (approx. 60 cm range) study area is in the north-central GOM at the terminus of the Mississippi and Atchafalaya rivers (Fig 1) located between 28.4^o^ to 29.7 ^o^ Deg. N and 89.5 ^o^ to 93.6 ^o^ W. The discharge from the Mississippi River watershed into the Gulf of Mexico averaged 21,367 ± 631 (μ ± 1 SE) cms from 1968 to 2016 (https://toxics.usgs.gov/hypoxia/mississippi/oct_jun/), which is a small volume compared to the continental shelf that is a 100 × 400 km wide and 100 m deep continental shelf off Louisiana. Approximately 30% of the Mississippi River joins with the Red River to form the Atchafalaya River. The combined discharges of the Mississippi and Atchafalaya rivers account for over 80% of the total freshwater input to the U.S. GOM [19]. Although there is high interannual variability in the timing of peak discharge, the mean high flow typically occurs in spring, with low flow in summer and fall. The Mississippi River empties onto a narrow (~20 km wide) continental shelf and there is no distinguishable river temperature signal offshore at the delta [20]. Its near-field plume separates rapidly from the bottom and expands laterally by buoyant spreading and entrainment. The Atchafalaya River, in contrast, empties onto the broad (~200 km) and shallow part of the shelf (< 100 m depth). The Atchafalaya River plume remains in contact with the bottom for more than 15 km seaward of the river mouth [21], and westward flow occurs in the Louisiana Coastal Current (LCC) throughout most of the year [22]. The mean flow during summer typically reverses and moves the Atchafalaya and Mississippi River waters to the east [21, 22]. The movement of the freshwater across the shelf at the surface in spring and summer ranges from 10 to 15 cm s^-1^ [21], and is <2 cm s^-1^ in bottom waters. The slowest water movement and the highest temperature and haline stratification is in the summer when winds are from the southeast and at their minimum to create upwelling favorable conditions [21, 22].

Cold fronts, tropical storms, and hurricanes typically cause enhanced alongshore and cross-shore transport, and a breakdown in water column stratification [21]. Tropical [23] and winter storm activity [24] is especially important in driving circulation patterns, salinity, and water level in the shallow estuarine outflow of the Atchafalaya River into the Atchafalaya Bay. Winds are weakest in the summer, except for aperiodic storm events disrupting the pycnocline (strongest in the summer) enough to temporarily re-oxygenate the water column when bottom-water salinity is at its seasonal high [21]. Freshwater is moved over the shelf in a matter of months that is attributed to advection, not evaporation [25]. The resulting balance of physical forces creates a surface layer that is mostly isolated from the bottom layer in summer, a weak down-welling/up-welling favorable cross-shelf movement, and also weaker shoreline-parallel bottom-layer movement. Summer is when there is a seasonal optimum for low-oxygen creation that is responsive to de-oxygenation resulting from the respiration of organic loading created in a eutrophic surface layer [26].

### Data sets

The earliest data set used is from Temple et al. [27] who made salinity and temperature measurements on the Louisiana-Texas continental shelf on 35 monthly cruises starting in January 1963 and ending December 1965 (Fig 1). They used a thermo-bathygraph to measure the complete temperature profile from surface to bottom-waters, and Nansen bottles to collect salinity measurements within 3 m of the bottom. Salinity was determined in the laboratory using a ‘Knudson’ method [28]. Dinnel and Wiseman [25] used these data to construct a freshwater budget for the nGOM shelf and to determine a 140 to 425 day freshwater fill time. We used the July data for 1963 to 1965 at stations overlapping with station locations of the second data set that spanned from 1985 to 2015. The 1963 to 1965 data were averaged by month and year for the same geographical area that was sampled in a more extensive effort during late July cruises (6–8 days each) beginning in 1985 and ending in 2015. This 1985–2015 systematic ‘shelfwide’ sampling program documented oxygen conditions on the shelf using the same station grid positions each year. The data are from 88 stations positioned at 5–15 km intervals along north-south transects whose east-to-west separation ranges from about 20 to 40 km apart (Fig 1). Water sampling used a hand-held instrument (various Hydrolab models and a YSI model 6820) to collect surface and bottom-water temperature and salinity measurements within 0.5 m below the surface and 0.5 m above the seabed, respectively. Salinity data were corrected as necessary by comparison to samples measured on an AutoSal or MiniSal. Data from all stations were not always sampled each year. There were no shelfwide cruises in 1989 or 2016.

We used only stations with greater than 8 years of data accounting for a total of 2,217 bottom-water temperature measurements, which yielded an average count of 67 data points for the summer of each year. The 1963–1965 data are in Temple et al. [27]. The 1985 to 2015 water temperature data were retrieved from the National Center for Environmental Information (formerly National Oceanographic Data Center; https://www.ncei.noaa.gov/archive, accession numbers: 9800129, 0002033, 0020956, 0032050, 0039733, 0049435, 0060060, 0069471, 0099531, 0017436, 0129417, 0162101, 0162440, 0161219, 164298).

The 1985–2015 data were divided into 5 depth zones: < 10 m, 10 to < 20 m, 20 to < 30 m, 30 to < 40 m, and between 40 and 63 m deep. The temperature, salinity and density at each station were averaged for each year for the surface and bottom-water. An ANOVA was run to test for differences between these depth-defined groups. We used the average monthly and average annual air temperature data for Louisiana Region 9 (15,960 km^2^) [29] located at the eastern half of the study area. We used a cross-correlation analysis to examine for a similarity between air and water temperatures as a function of the lag of one relative to the other to determine how long it might take for the heat from the atmosphere to enter the bottom layer.

The subsets of data tested included the monthly average air temperature for the 5 spring months before the cruises (March, April, May, June and July), and different combinations of months. We used a Prism 6 (ver. 6) statistical package (Software, Inc.; www.graphpad.com) to conduct linear regression analyses, and to test for differences in regression slopes, and to test for a trend in the residuals.

## Results

**Fig 2:**
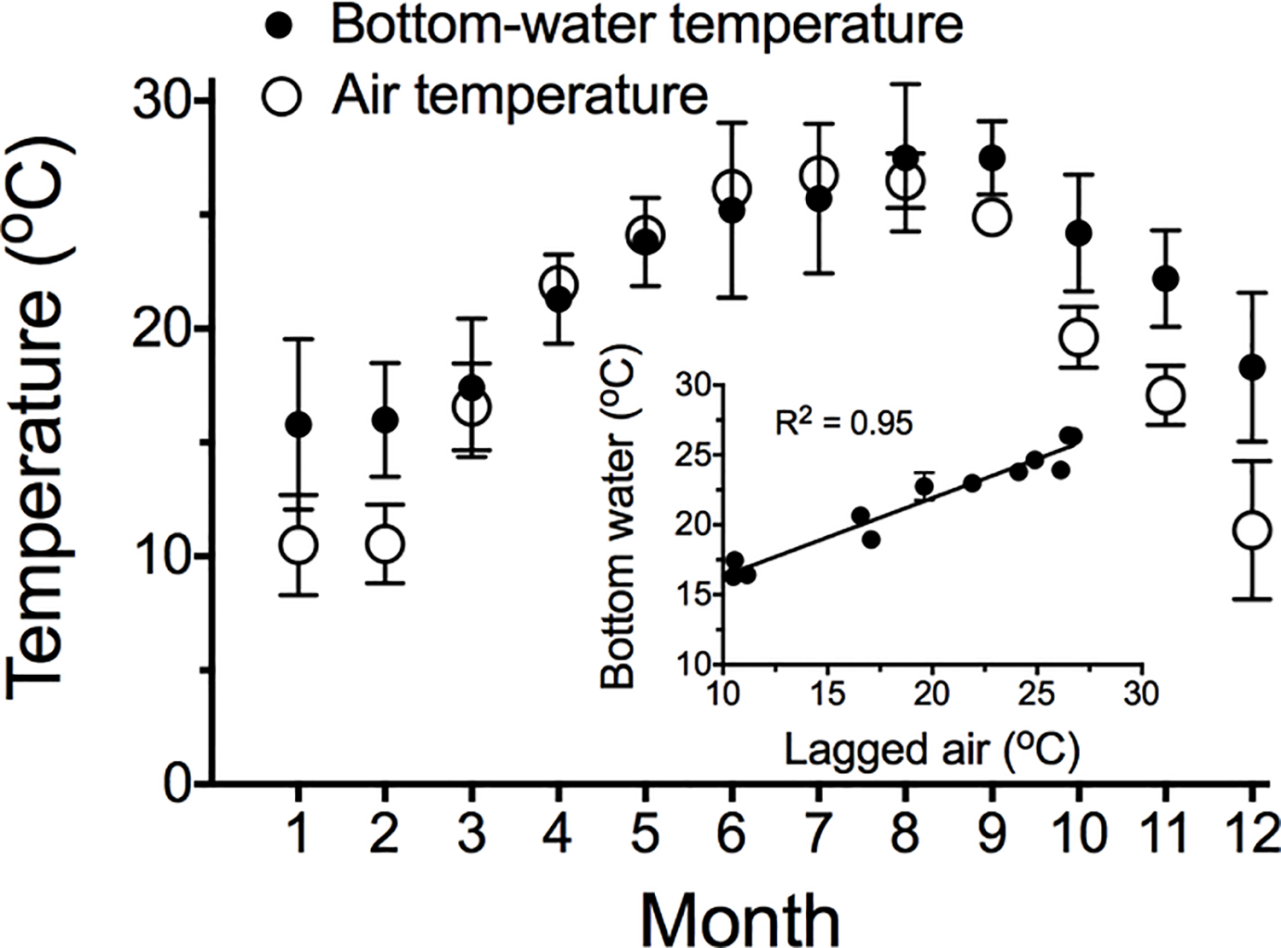
The monthly average temperature for the continental shelf bottom-water and for air temperature at the New Orleans airport for 1963–1965. The insert is a linear regression of bottom-water temperature vs. air temperature lagged by one month.

The 1963–1965 data showed a seasonal rise and fall of bottom-water temperature that was coincidental with the air temperature, although bottom-water heating and cooling lagged air temperature by one month (Fig 2). The bottom-water temperature peaked in August at approx. 26 ^o^C, when it was equal to the maximum air temperature, and fell to around 16 ^o^C in January and February. The annual average winter bottom-water temperature was about 5 ^o^C warmer than the air temperature.

**Fig 3:**
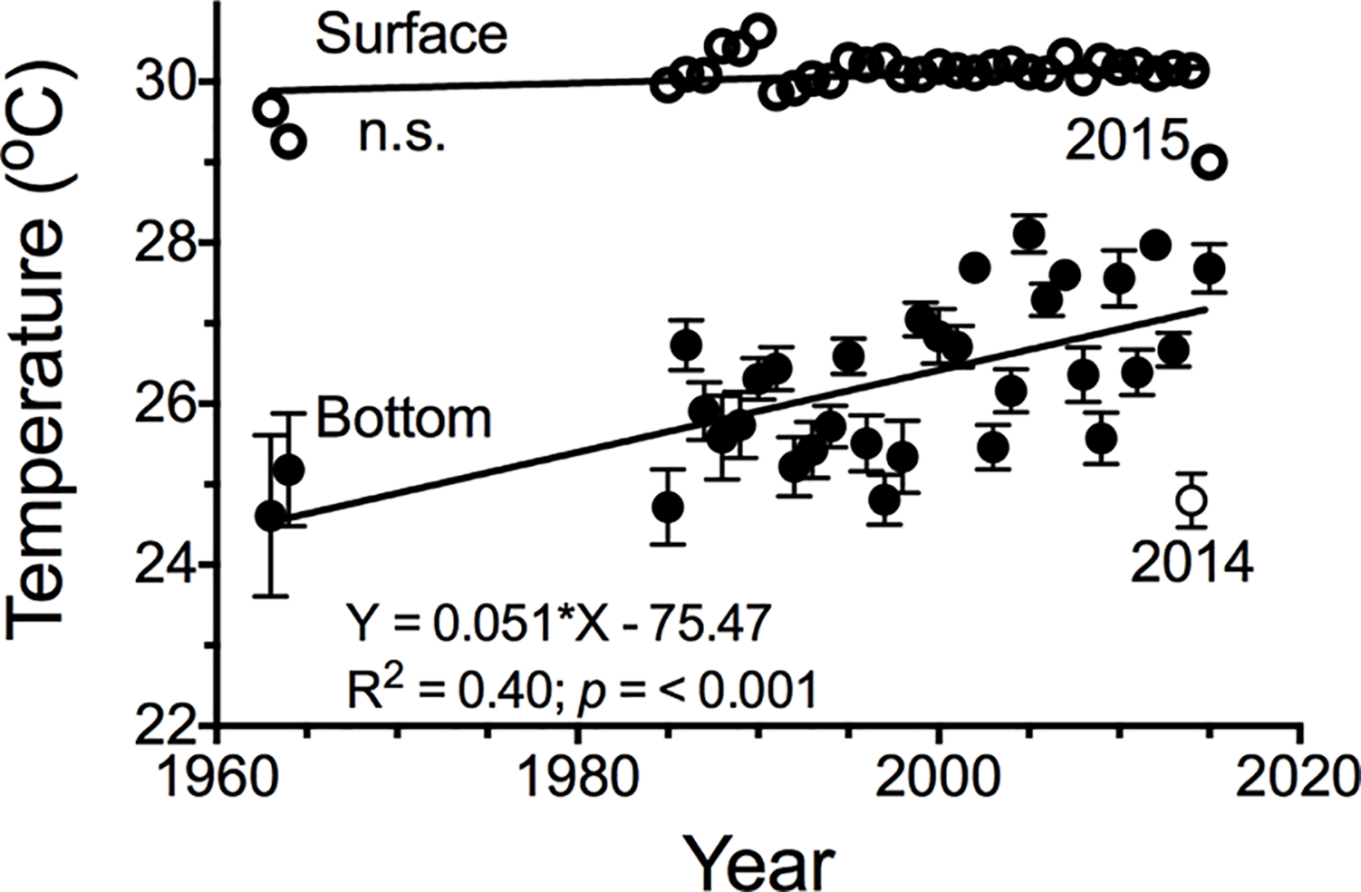
Annual average temperature for surface and bottom-waters in late summer. A linear regression of bottom-water temperature and year excludes the 2014 data. Vertical error bars denote ± 1 standard error.

The average temperature of all bottom-water samples increased by 0.044 ± 0.012 ^o^C y^-1^ (μ ± 1 SE) from 1963 to 2015 (*p* < 0.001), but there was no statistically-significant increase in the surface water temperature (Fig 3). The average temperature for 2014 appears as an outlier in these plots, and excluding it from the analysis raises the slope to 0.051± 0.011 ^o^C y^-1^.

**Fig 4:**
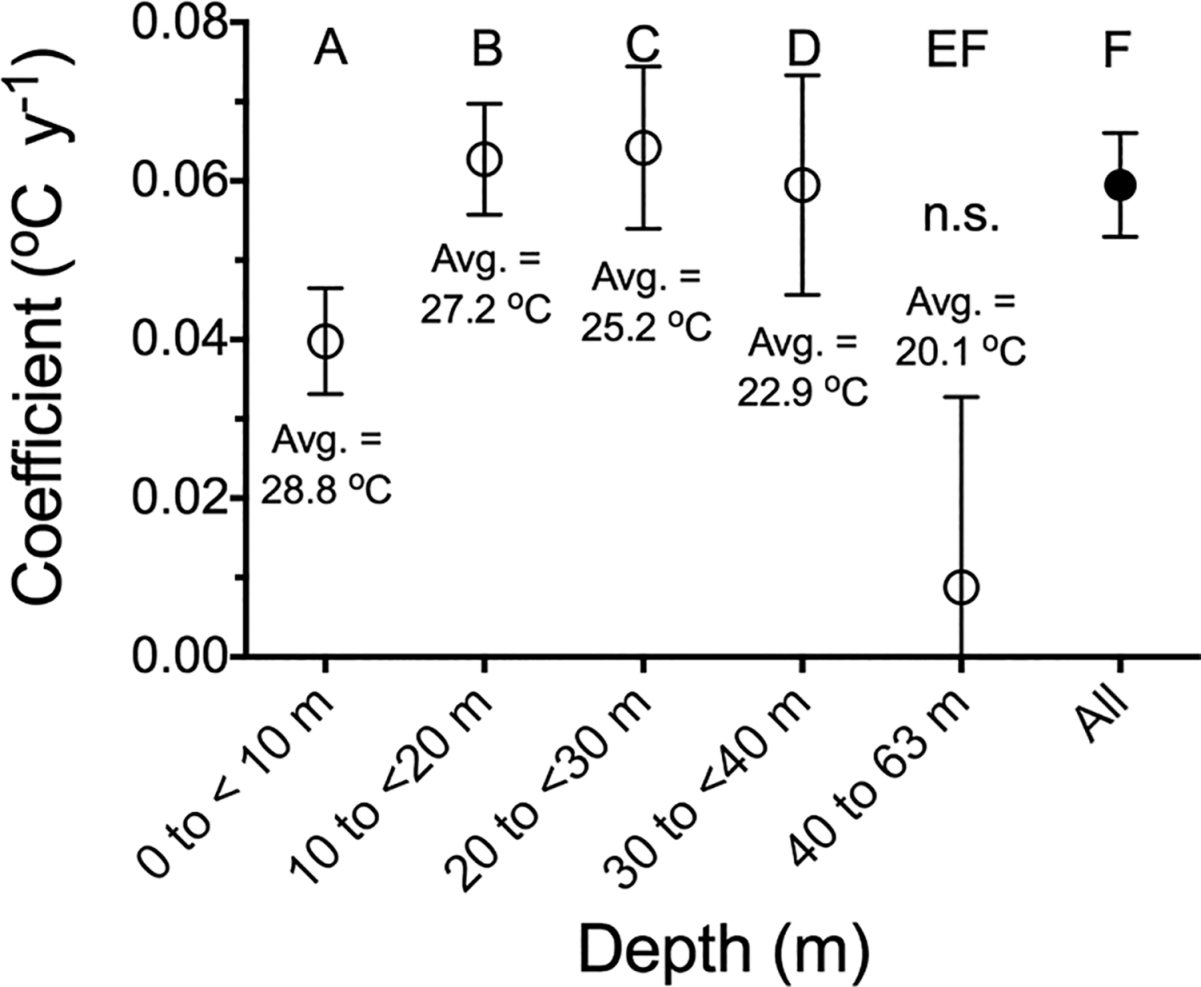
The annual increase in bottom-water temperature from 1985 to 2015 for five bottom-water stations grouped by depth zones. The coefficient is the slope from a linear regression of year vs. temperature (μ ± 95% CI). Different letters represent statistically-significant differences between data groups.

The bottom-water temperature increase for the 1985 to 2015 data was lowest for the shallowest (<10 m) and deepest (>40 to 63 m) depth zones (Fig 4; Figure A in S1 File. The average temperature increase in the water <10 m deep was about one-half of the increase at intermediate depths. The temperature variations at the deepest stations (<40 to 63 m) were too high to detect a trend (Fig 4). It is perhaps instructive to note that the average bottom-water temperature for the shallowest stations had the highest temperature.

**Fig 5:**
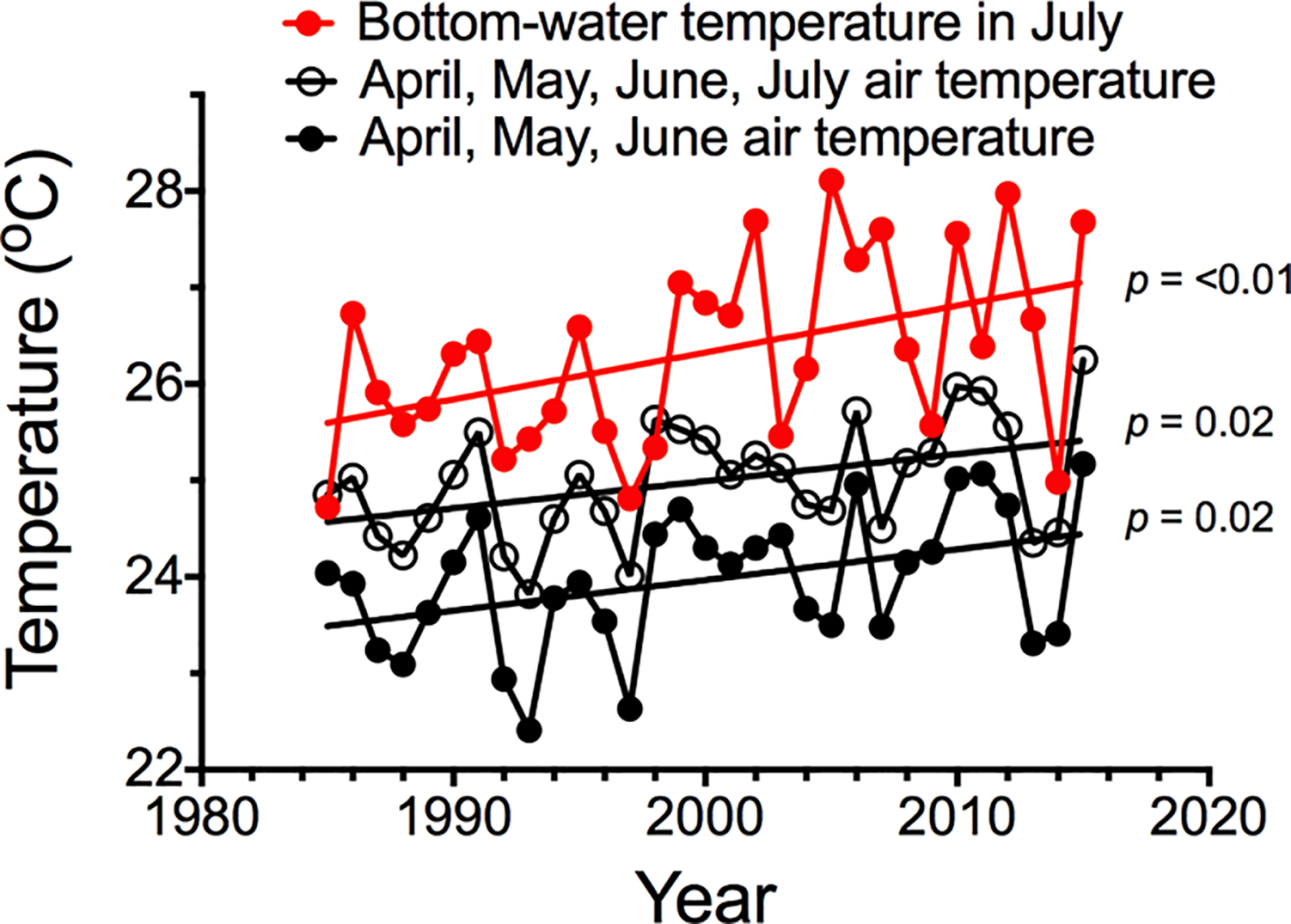
Trends in the annual average July bottom-water temperature and air temperatures for two different combinations of monthly averages from 1985 to 2015.

The fluctuations in summer bottom-water temperature from 1985 to 2015 are somewhat, but not completely, synchronous with fluctuations in air temperature for the preceding spring months (Fig 5). Slight changes in the spring air temperature were coincidental with changes in the bottom water temperatures, and had a coefficient of determination (R^2^) value of 0.26 to 0.28 (*p* < 0.01) for the various combinations of air temperature from April to July. There were no statistically-significant differences in the slopes of the two monthly combinations vs. year. The temperature trends for the average July air temperature and the summer bottom-water temperature from 1963 to 2015 were 0.028 ± 0.011 and 0.044 ± 0.012 ^o^C y^-1^ (μ ± 1 SE), respectively, both of which were statistically significant (*p* < 0.01), but the slopes were not significantly different from each other. A plot of the residuals of the linear regressions for bottom temperature or lagged air temperature vs time (Figure E in S1 File) demonstrated no trend that would violate the assumptions of linear regression, and so no further tests were made to detect autoregressive covariance.

**Fig 6:**
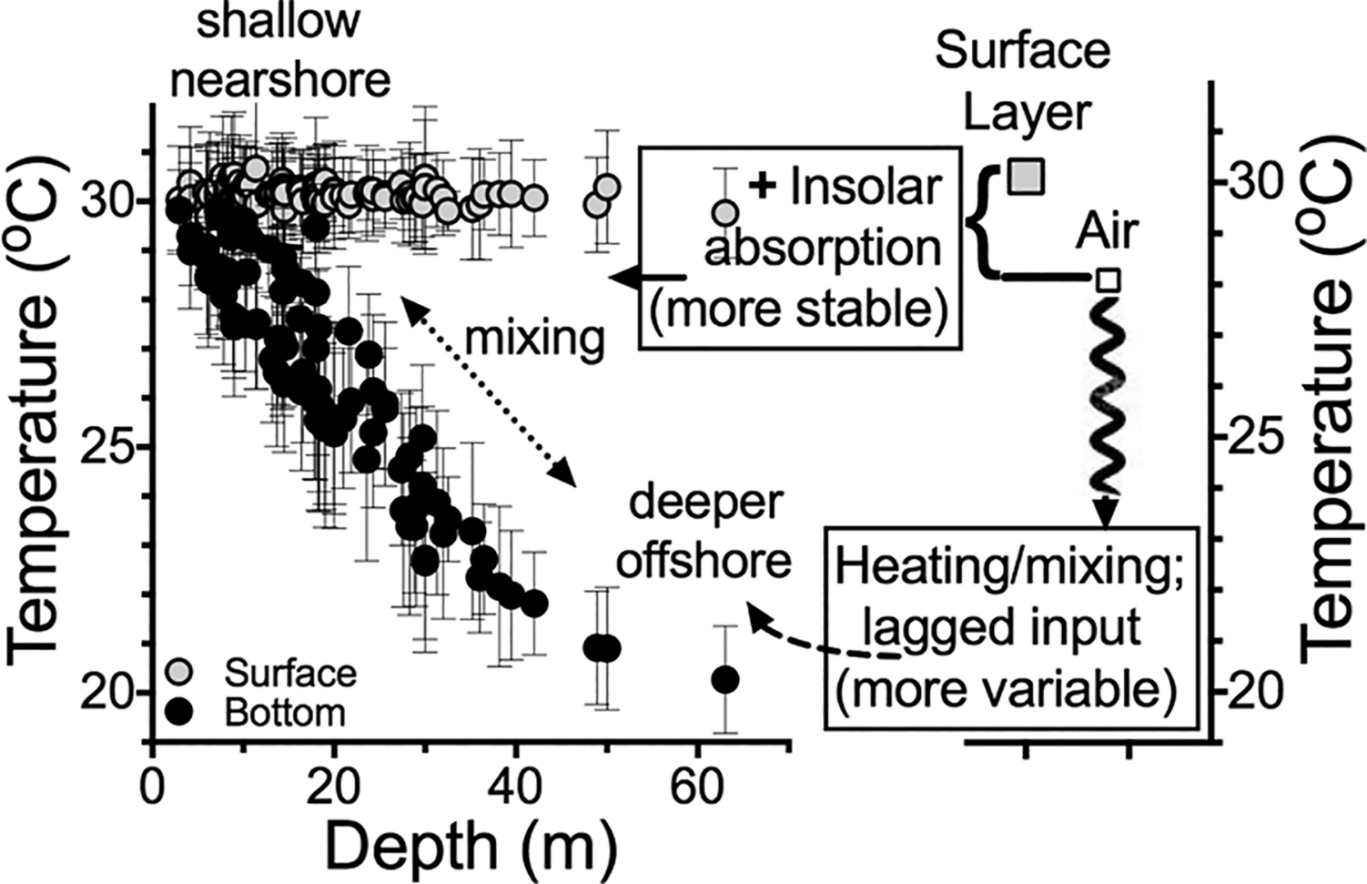
The relationship between station depth and the average summer bottom-water temperature at the 88 stations shown in Fig 1 for the period 1985–2015.

The 1985 to 2015 average summer bottom-water temperature had about a 10 ^o^C gradient over the 60 m depth range (Fig 6) whose coefficient of variation for the 1,887 samples was 10.0%. The coefficient of variation for the 30-year average surface water temperature was 0.86%. The corresponding coefficient of variation for monthly air temperatures from March to July ranged from 2 to 10%. The coefficient of variation for only July air temperature was 1.93%. The July summer surface-water temperature, in other words, was much more stable among years than the spring monthly air temperature, which was higher than the variance in summer bottom temperature. There was no detectable change from 1985 to 2015 for surface and bottom salinity values (Figure B in S1 File), water column stability (Figure C in S1 File), or the relationships between temperature and salinity in surface and bottom samples (Figure D in S1 File).

## Discussion

The bottom-water temperature on this shelf varies seasonally with changes in air temperature, and the variations from year-to-year are dis-proportionately larger in the bottom layer compared to the surface layer. The gradual decline with depth belies the east to west homogeneity in the factors driving the movements of bottom-water across the shelf in a north to south direction. This result supports, but does not prove, a conclusion that the summertime variations in the bottom-water temperature on this shelf are controlled more by the intrusions of deeper GOM water onto the shelf than the influence of the Mississippi and Atchafalaya rivers at the eastern margin. The summertime warming in bottom-water, however, is 1.6 times faster than the corresponding summertime warming in regional air temperature, and the surface water temperature shows no significant trend over time.

There are differences in the summertime temperature trends for bottom and surface layers, as well as in the air, because, in part, land and turbid water warms up and retains heat more efficiently than air. The heat capacity of clear water at 25 ^o^C, for example, is about four times that of air, and even higher for turbid waters. The heat absorption is higher, therefore, in turbid waters in shallow depths [30, 31]. The resulting latent heat storage in the air is much more variable because of the greater thermal inertia of oceans, and so there is a legacy effect of the warming in the open ocean seen in the vertical temperature profiles [1,5].

The different temperatures in air and water reflect the source trajectories on this shelf. The temperature of the bottom layer on the shelf break at >100 m is dependent on the mixing of cooler and saltier water from upwelled water at the shelf break onto the shelf, and also because the alongshore summer current velocity on the shelf is at its seasonal minimum, especially below the pycnocline [30]. The temperature of this offshore water varies from the effects of fluctuating wind speed, evaporative cooling, downward mixing, and slow upward movement onto the shelf. The result of these influences is that the bottom-water temperature is strongly related to the past air temperatures integrated over several months (Fig 5). It is cooler than the offshore surface water which, at depths of a few hundred meters, is mixed with cooler and deeper water before it begins its shelfward ascent. The lower variability in the summertime heat content of the surface layer, therefore, reflects the stable solar input rather than evaporative cooling, and also the meager mixing with deeper and cooler water.

The heat balance is determined by the air temperature and oceanic sources, but not from the estuaries or river for the following reasons: 1) A gross heat budget for the northern GOM does not consider river heat inputs as a significant heat source in their model [31]; 2) The heat island at the Mississippi River mouth is inconsequential–the temperature in the river is not monitored satisfactorily, but is close to air temperature at the mouth of the River Delta [25]; 3) The volume of river water is about 10% of the volume of the Louisiana shelf west of the Mississippi Delta and has a freshwater fill time of 3 months [25]; 4) there is no east to west gradient in the bottom temperature; 5) there is no significant physical mechanism in effect to transfer heat from the estuaries to shelf; 6) the freshwater river debouching at the delta headwaters is the surface layer of a stratified system–which contributes to the hypoxia (<2 mg L^-1^) formed most summers [26].

There are several significant consequences of the bottom-water temperature increase. One is that it partially explains the coefficient for the year of sampling in a model predicting the extent of the summertime low-oxygen zone on this shelf [32]. This ‘Year’ coefficient is independent of a nutrient loading effect, and represents part of a greater sensitivity of hypoxia to riverine nutrient loading [33]. The effect could be a result of greater metabolic demand in response to higher temperatures in water and sediments. The sediment oxygen demand could be as much as 73% of the total respiratory demand of the water column on this shelf [34]. Also, oxygen saturation is lower at higher temperatures. A 2 ^o^C increase at 30 psu, for example, decreases oxygen saturation by 3%. Importantly, the density stratification has remained relatively unchanged from 1985 to 2015 (Figure C in S1 File), whereas the hypoxic area expanded substantially [33]. There is no demonstrable change in summertime water column stability among years because variations in surface salinity, not temperature, have the controlling influence on density stratification. This absence of a change in density stratification supports the conclusion that the increased areal extent and severity of hypoxia over time is driven by increased nutrient loading, but not from increased water column stability as a result of warming.

Altieri and Gedan [35] used air temperature to predict a 2 ^o^C increase in water temperature for the world’s hypoxia zones by the end of this century. They argued that such an increase would be a significant driver of bottom-water hypoxic water mass formation, size, duration and effects. The rise of a current bottom-water temperature increase of 2 ^o^C on the GOM shelf over the last 30 years is equivalent to their global estimate for surface waters. Their prediction for an additional 2 ^o^C temperature increase on the GOM shelf for this century seems conservative, therefore, given that the bottom-water temperatures have been rising 2.8 times faster than air temperature, and that atmospheric warming is expected to accelerate in the future [36].

The annual air temperature at New Orleans, Louisiana, increased at 0.034 ^o^C y^-1^ from 1985 to 2015, or about 2.7 times the rise in annual global sea surface temperature averaged for the open GOM (0.0129 ^o^C y^-1^) over the same time [37]. The extremes in regional air temperature for the southeast US have been increasing from 1948–2012 [38]. The overall increase in the summertime bottom-water temperatures on the nGOM shelf is, therefore, 1.9 times faster than the annual air temperature warming, which itself was 0.046 / 0.0097 = 4.7 times faster that the global ocean temperature increase from 1998 to 2014 (0.0097 ^o^C y^-1^) [39].

The rate of temperature rise in bottom-waters (1985–2015; 0.046 ^o^C y^-1^) can be compared to a few measurements reported for the GOM and other coastal regions. The bottom-water temperature increase of the European continental shelf (depths 5–592 m, average water temperature ~ 8 ^o^C) from 1980 to 2008 was 0.04 ^o^C y^-1^ [8]. White and Visser [40] estimated that the average annual temperature of the Mississippi water within the Bird Foot’s delta rose at 0.09 ^o^C y^-1^ from 1983 to 2012. We find no major temperature signature of the river in the offshore surface water that is more than a few kilometers from the delta [41], which suggests that the river has a minimal influence on offshore water temperatures west of the delta. Fodrie et al. [42] found that the water temperature in the northwestern GOM shallow water sea grass beds increased by 0.10 ^o^C y^-1^ from 1987 to 2007. The corresponding temperature increased by 0.0281 ^o^C y^-1^ from 1975 to 2007 in the upper 4 m of water in the Florida Keys, and was 0.0281 ^o^C y^-1^ from 1975 to 2007 [43]. The baywide temperature of eight Texas estuaries rose by 0.0428 ^o^C y^-1^ from 1976 to 2007 [44]. The bottom-water temperature rise rates on this shelf are, therefore, significantly large for the region and well above the global average for the surface waters.

Although the volume of water on the continental shelves is small relative to the water mass in the oceans, the shelves are a site of major biologically-intense functions. It would be interesting to see how the depth profiles of cumulative temperature content change over time, and how much these changes account for the changes in heat budget of the entire GOM. Continuing the long-term temperature sampling program would be an effective and prudent action to evaluate future trends and consequences.

## Supporting information

S1 File **Figure A in S1 File. The temperature values for each bottom-water sample for five depth zones from 1985 to 2015 for summertime values**. **Figure B in S1 File. The change in the average summer salinity values in bottom and surface samples from 1985 to 2015**. The trends in the bottom and surface salinities are not statistically significant. **Figure C in S1 File. The change in the average summer density difference between bottom and surface samples from 1985 to 2015**. The trends in the delta sigma t values are not statistically significant. **Figure D in S1 File. The relationships between the average summer temperature and salinity values for bottom and surface samples**. **Figure E in S1 File. The residuals of the plot of the linear regression for the data in Figs 3 and 5 for the bottom water temperature vs. year (upper) and lagged air temperature (bottom).**
(DOCX)
