# Supplementary material for: Trends in summer bottom-water temperatures on the northern Gulf of Mexico continental shelf from 1985 to 2015
Source: PLoS One. 2017 Sep 7;12(9):e0184350. doi: 10.1371/journal.pone.0184350 (PMC5589215; doi:10.1371/journal.pone.0184350)
Supplement: S1 File — Figure A in S1 File. The temperature values for each bottom-water sample for five depth zones from 1985 to 2015 for summertime values. Figure B in S1 File. The change in the average summer salinity values in bottom and surface samples from 1985 to 2015. The trends in the bottom and surface salinities are not statistically significant. Figure C in S1 File. The change in the average summer density difference between bottom and surface samples from 1985 to 2015. The trends in the delta sigma t values are not statistically significant. Figure D in S1 File. The relationships between the average summer temperature and salinity values for bottom and surface samples. Figure E in S1 File. The residuals of the plot of the linear regression for the data in Figs 3 and 5 for the bottom water temperature vs. year (upper) and lagged air temperature (bottom). (DOCX) [file pone.0184350.s001.docx]

**Supplemental Materials**

Bottom-water Temperature in Summer on the Northern Gulf of Mexico

Continental Shelf from 1985 to 2015

R. Eugene Turner^1,* ¶^, Nancy N. Rabalais^1,2¶^, Dubravko Justić^1¶^

^1^ Department of Oceanography and Coastal Sciences, Louisiana State University, Baton Rouge, Louisiana 70803 USA

^2^ Louisiana Universities Marine Consortium, Chauvin, Louisiana 70344 USA

**Figure A in S1 File.** **The temperature values for each bottom-water sample for five depth zones from 1985 to 2015 for summertime values**.

**Figure B in S1 File.** **The change in the average summer salinity values in bottom and surface samples from 1985 to 2015**. Trends in the bottom and surface salinities are not statistically significant.

**Figure C in S1 File.** **The change in the average summer density difference between bottom and surface samples from 1985 to 2015**. Trends in the delta sigma t values are not statistically significant.

**Figure D in S1 File.** **The relationships between the average summer temperature and salinity values for bottom and surface samples**.

**Figure E in S1 File. The residuals of the plot of the linear regression for the data in Fig. 3 and 5 for the bottom water temperature vs. year (upper) and lagged air temperature (bottom).**


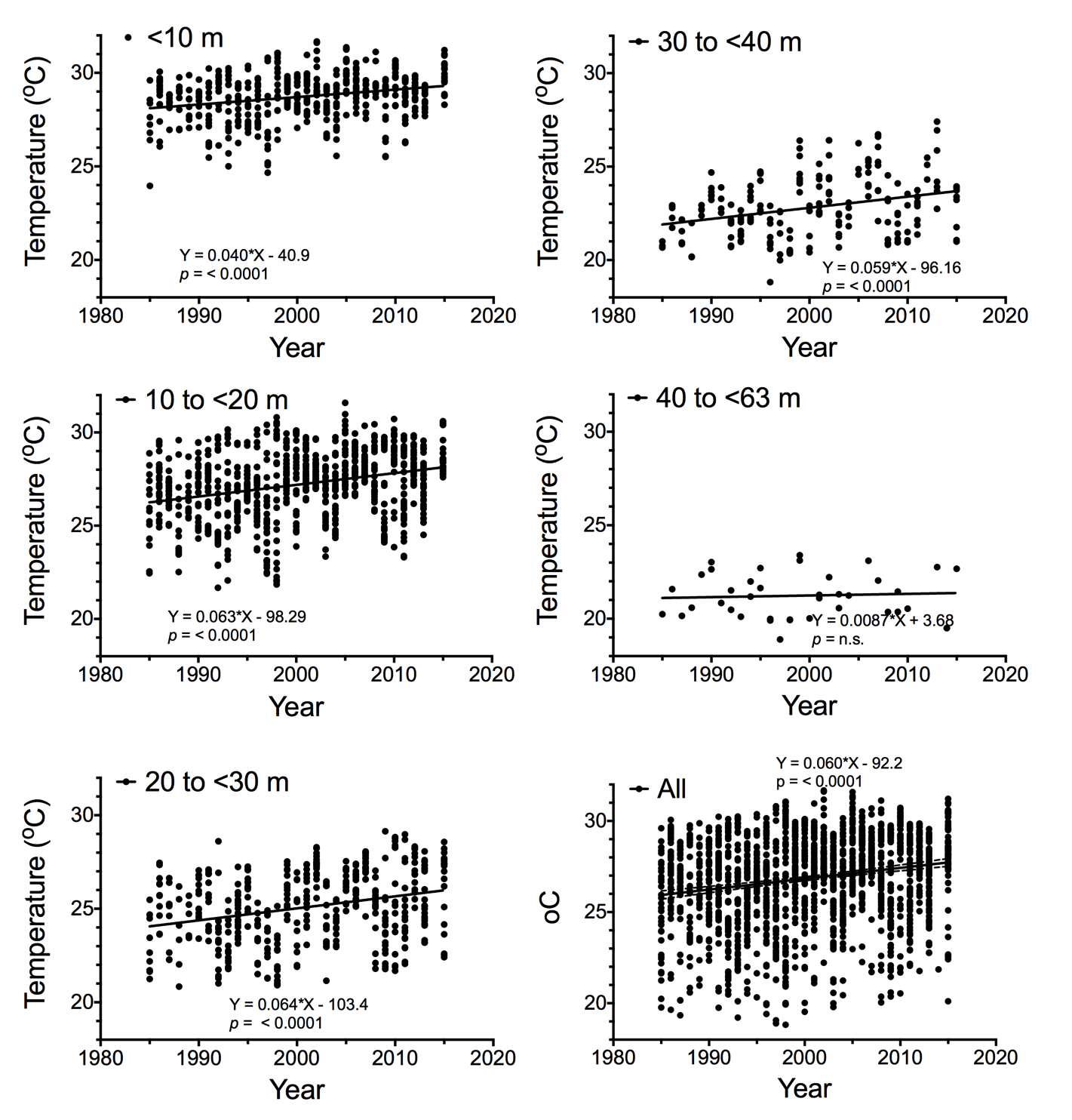


**Figure A in S1 File.** **The temperature values for each bottom-water sample for five depth zones from 1985 to 2015 for summertime values**.


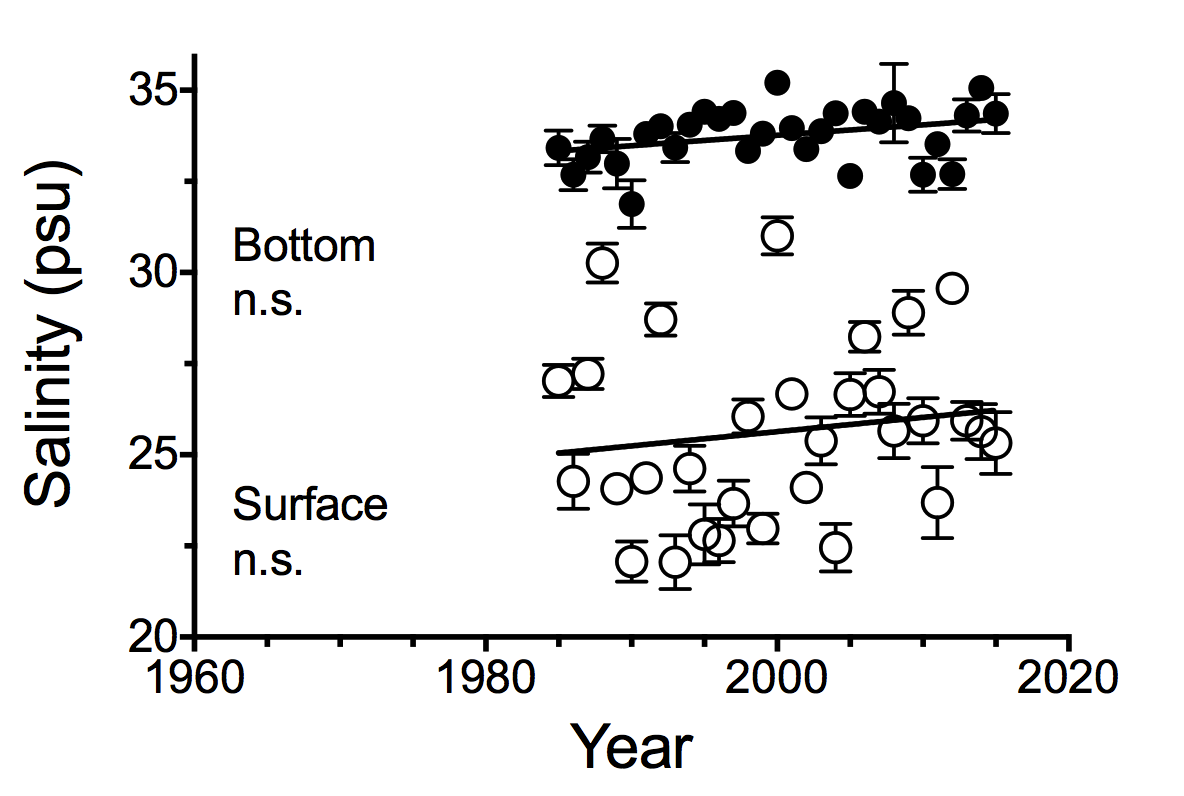


**Figure B in S1 File.** **The change in the average summer salinity values in bottom and surface samples from 1985 to 2015**. Trends in the bottom and surface salinities are not statistically significant.


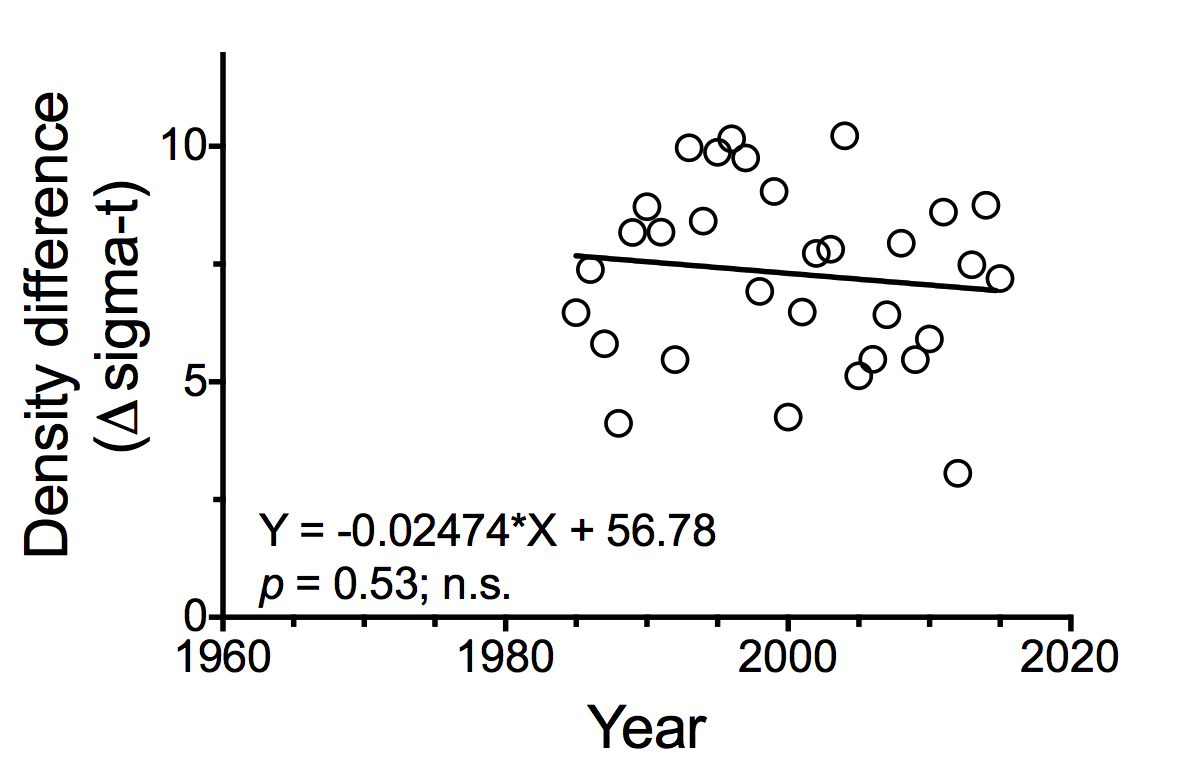


**Figure C in S1 File.** **The change in the average summer density difference between bottom and surface samples from 1985 to 2015. Trends in the delta sigma t values are not statistically significant**.


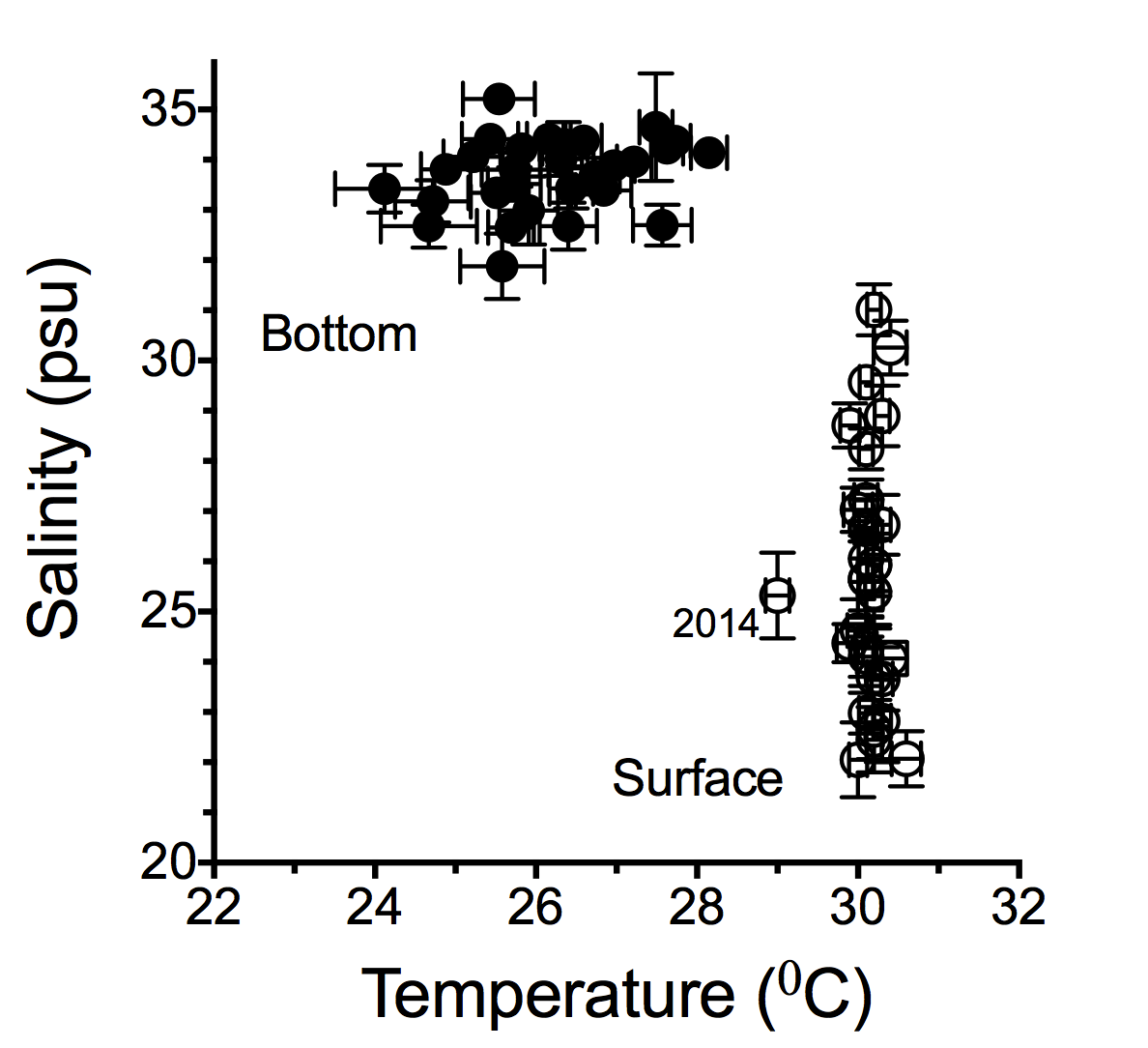


**Figure D in S1 File.** **The relationships between the average summer temperature and salinity values for bottom and surface samples**.


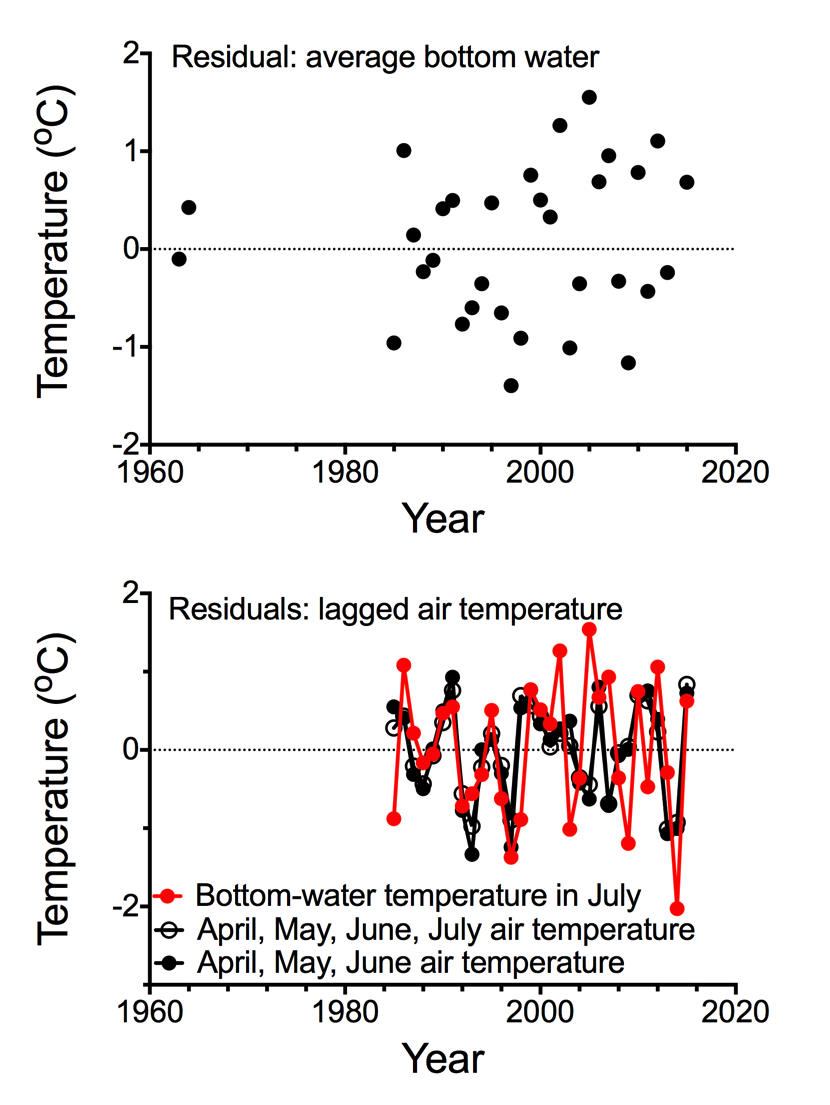


**Figure E in S1 File. The residuals of the plot of the linear regression for the data in Fig. 3 and 5 for the bottom water temperature vs. year (upper) and lagged air temperature (bottom).**
